# Phylogenetic, Structural, and Evolutionary Insights into Pepper NBS-LRR Resistance Genes

**DOI:** 10.3390/ijms26051828

**Published:** 2025-02-20

**Authors:** Jia Liu, Yuan Cheng, Meiying Ruan, Qingjing Ye, Rongqing Wang, Zhuping Yao, Guozhi Zhou, Chenxu Liu, Hongjian Wan

**Affiliations:** 1 State Key Laboratory for Quality and Safety of Agro-Products, Zhejiang Academy of Agricultural Sciences, Hangzhou 310021, China; liu-l-jia@163.com; 2 Institute of Vegetables, Zhejiang Academy of Agricultural Sciences, Hangzhou 310021, China; chengyuan@zaas.ac.cn (Y.C.); ruanmy@zaas.ac.cn (M.R.); yeqj@zaas.ac.cn (Q.Y.); wangrq@zaas.ac.cn (R.W.); yaozp@zaas.ac.cn (Z.Y.); zhougz@zaas.ac.cn (G.Z.); liuchenxu@zaas.ac.cn (C.L.)

**Keywords:** Capsicum, NBS-LRR, nTNL, TNL, gene clusters, tandem duplication

## Abstract

The comprehensive analysis of NBS-LRR resistance genes in the pepper (*Capsicum annuum* L.) genome reveals their structural diversity, evolutionary history, and functional importance in plant immunity. A total of 252 NBS-LRR genes were identified, distributed unevenly across all chromosomes, with 54% forming 47 gene clusters. These clusters, driven by tandem duplications and genomic rearrangements, underscore the dynamic evolution of resistance genes. Phylogenetic analysis demonstrated the dominance of the nTNL subfamily over the TNL subfamily, reflecting lineage-specific adaptations and evolutionary pressures. Structural analyses identified six conserved motifs (P-loop, RNBS-A, kinase-2, RNBS-B, RNBS-C, and GLPL) essential for ATP/GTP binding and resistance signaling. Subfamily-specific differences in motif composition and sequence similarity highlight their functional divergence and specialization. Comparative analyses across species further revealed a greater prevalence of nTNL genes in angiosperms, with significant losses of TNL genes in monocots. This study enhances our understanding of the evolution and diversification of plant-resistance genes and provides a foundation for developing disease-resistant crops through targeted breeding strategies.

## 1. Introduction

Disease resistance is a critical trait in plant breeding, as plants face invasion by a wide variety of pathogens, including bacteria, fungi, oomycetes, viruses, and nematodes. Susceptibility to these pathogens can severely limit plant growth and yield, as exemplified by bacterial spot disease in pepper caused by *Xanthomonas campestris* and late blight in potato caused by *Phytophthora infestans* [1,2,3,4]. To combat these threats, plants have evolved highly effective mechanisms to recognize and defend against pathogens, with disease resistance (R) genes playing a pivotal role. Among the more than 140 characterized R genes, approximately 80% encode proteins containing Nucleotide-Binding Site (NBS) and Leucine-Rich Repeat (LRR) domains, which provide resistance to a wide range of pathogens [5,6].

NBS-LRR genes represent the largest class of R genes, contributing to both pathogen recognition and downstream defense signaling [7]. Within these resistance proteins, the NBS domain is highly conserved and plays a critical role in ATP and GTP hydrolysis and binding, which are essential for initiating immune signaling [8]. The LRR domain, known for its involvement in protein–protein interactions, is believed to be responsible for recognition specificity [9,10] between the NBS and LRR domains, the ARC (auxiliary) domain functions to recruit the LRR domain and regulate the protein’s inactive and active states [11]. According to the guard hypothesis, NBS-LRR proteins monitor plant targets for pathogen effector proteins, becoming activated upon interaction between guard proteins and virulence factors [12,13].

The typical structure of an NBS-LRR resistance gene includes three main domains: a Toll/Interleukin-1 receptor (TIR) or coiled-coil (CC) domain at the N-terminus, an NBS domain in the middle, and an LRR domain at the C-terminus [14,15,16,17,18]. Based on differences in their N-terminal domains, NBS-LRR resistance genes are classified into two subclasses: TIR-NBS-LRR (TNL) and CC-NBS-LRR (CNL), also referred to as non-TIR-NBS-LRR (nTNL) [15,19]. The CC domain facilitates protein–protein interactions, while the TIR domain is involved in signal recognition and transduction. The NBS domain, containing several conserved motifs of 10–30 amino acids, is crucial for signal initiation [6,20]. In contrast, the LRR domain is highly variable, enabling pathogen-specific recognition [9,10,14,21].

The conserved sequences within the NBS domain are widely used to identify and isolate plant resistance gene analogs (RGAs). Primers are designed based on conserved regions of the NBS domain to amplify RGAs, which are then mapped to chromosomes using molecular markers and genetic maps. Over time, resistance genes are identified through this process. For example, previous researchers successfully designed probes targeting the NBS domain to identify resistance genes in grapes [22]. With the advent of whole-genome sequencing, the identification of NBS-LRR resistance genes and corresponding cDNA sequences has become more efficient and widespread. Gene clusters of NBS-LRR genes are commonly formed through tandem duplications, with genes in the same cluster sharing high sequence similarity and fixed chromosomal locations. Using these characteristics, researchers have successfully identified gene clusters and developed molecular markers [23].

Pepper (*Capsicum annuum* L.), one of the most widely cultivated vegetable crops globally, has the largest cultivated area in China. However, pepper is highly susceptible to various diseases, including phytophthora blight, powdery mildew, and root-knot nematodes, which cause significant production losses. The conserved sequences of NBS-LRR resistance genes provide an effective means of isolating and cloning resistance genes in pepper, aiding in studies of its defense mechanisms, genetics, and evolutionary history. Understanding the domains, clustering patterns, and evolutionary relationships of NBS-LRR resistance genes is essential for advancing pepper breeding programs and improving resistance to pathogens.

## 2. Results

### 2.1. Structural and Phylogenetic Characterization of NBS-LRR Resistance Genes in the Capsicum Genome: Diversity, Subclassification, and Evolutionary Insights

BLAST and HMM searches identified a total of 252 NBS-LRR resistance genes from the pepper genome. These genes were further analyzed using Pfam and COILS to identify CC, TIR, and LRR domains. Based on the presence or absence of a TIR domain at the N-terminus, all 252 NBS-LRR resistance genes were categorized into two main groups: 248 nTNLs (non-TIR NBS-LRR) and 4 TNLs (TIR NBS-LRR). Among these, only 48 genes were found to contain a CC domain, two of which were identified as typical CNL genes within the nTNL subfamily. Interestingly, 200 of the identified genes lacked both CC and TIR domains, further emphasizing the diversity of NBS-LRR resistance genes in the Capsicum genome (Table 1).

Using Pfam identification, the nTNL genes were classified into six subclasses based on their domain structure: (1) N, which contains only the NB-ARC domain; (2) NL, consisting of NB-ARC and LRR_8 domains; (3) NLL, composed of NB-ARC and two LRR_8 domains; (4) NN, containing two NB-ARC domains; (5) NLN, consisting of NB-LRR and NB-ARC domains; and (6) NLNLN, comprising NB-LRR, NB-LRR, and NB-ARC domains. The TNL genes, in contrast, were assigned to a single subclass: TN, which includes TIR and NB-ARC domains (Table 1). Notably, among the 252 NBS-LRR resistance genes, the NLNLN subclass is represented by only one gene, making it the rarest among all subclasses. For phylogenetic analysis, a subset of 111 conserved NBS domain sequences were selected. These sequences represent a core group of genes with significant structural conservation, providing valuable insights into the evolutionary relationships within the NBS-LRR gene family. This classification and phylogenetic study highlight the structural and functional diversity of resistance genes in pepper, laying a foundation for future research into their roles in plant defense and their potential applications in crop improvement.

### 2.2. Genomic Distribution, Diversity, and Clustering of NBS-LRR Resistance Genes in Capsicum: Implications for Functional Evolution

The identified NBS-LRR resistance genes are distributed across all chromosomes in the Capsicum genome, although their numbers vary across individual chromosomes. Notably, 31 genes are located on an unassigned chromosome, referred to as chromosome 0. These genes on chromosome 0 are exclusively NB-ARC genes, which constitute the largest proportion of NBS-LRR genes in the genome. All pepper chromosomes contain NB-ARC genes, highlighting their ubiquitous distribution. Among the 12 identified chromosomes, chromosome 3 harbors the highest number of genes, totaling 38, while chromosomes 2 and 6 each contain the lowest number, with only 5 genes. Chromosome 12 stands out due to its high diversity of gene subfamilies, encompassing five categories: TN, NL, NN, NLN, and N. In contrast, the NLNLN gene subfamily, represented by only one gene, is located on chromosome 9, making it the rarest subfamily among the identified genes.

**Figure 1:**
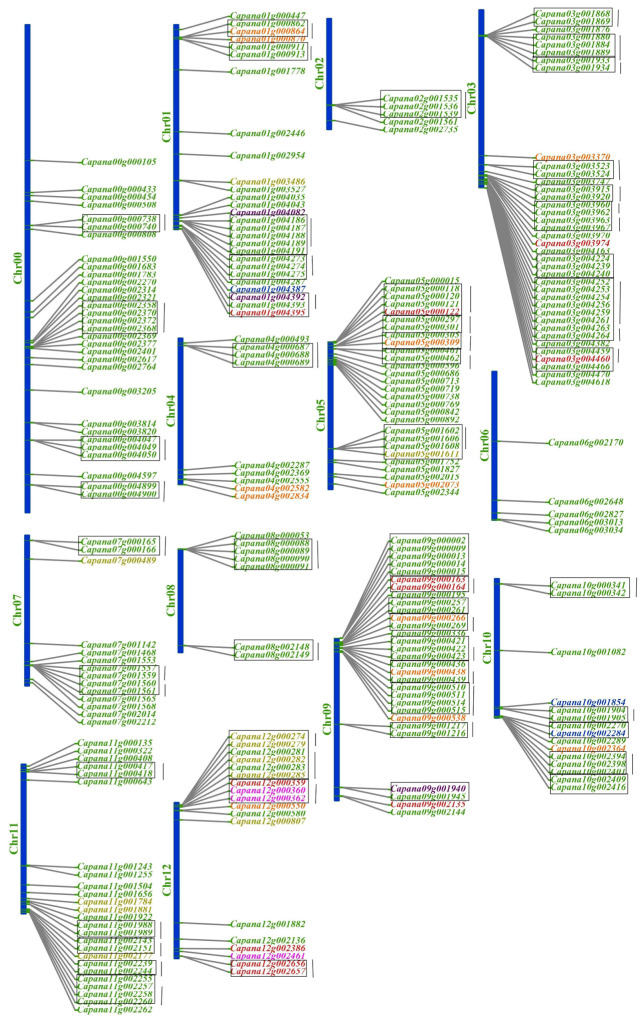
Chromosomal distribution of NBS-LRR resistance genes in the pepper (Capsicum annuum) genome. The figure illustrates the locations of NBS-encoding genes across all 12 chromosomes and an unassigned chromosome (Chr. 0). Genes are categorized into subclasses based on their domain compositions, represented by different colors: N (green), NN (orange), NL (red), NLL (blue), NLN (yellow), NLNLN (purple), and TN (magenta). Genes within the same subclass are grouped together. Gene clusters are delineated by black brackets, and singleton genes are mapped as isolated entities. Chromosome 12 displays the highest diversity of gene subclasses, while chromosome 6 has the fewest genes and lacks clusters. The distribution highlights the uneven localization and clustering patterns of resistance genes across the pepper genome.

The genetic linkage map was generated using MapDraw (Figure 1) to provide a clear visualization of the gene distribution. To enhance clarity, genes from the same subfamily are represented in the same color. Chromosomes 0, 2, and 6 exclusively contain NB-ARC genes, indicating a specialized distribution pattern. Chromosome 12, with its diverse gene subfamilies, is a hotspot for genetic diversity in the NBS-LRR resistance gene family. Interestingly, an RPW8 NB-ARC (RN) gene was also identified on chromosome 4. RPW8 genes, as reported in Arabidopsis, are known to confer broad-spectrum resistance to powdery mildew [15]. According to the definition of gene clusters (see Section 4 “Materials and Methods”), 136 NBS-LRR resistance genes are physically clustered within the genome, representing 54% of the total identified genes and forming 47 distinct gene clusters. Each chromosome contains varying numbers of gene clusters, with the highest number (10 clusters) located on chromosome 3. The largest gene cluster, comprising eight genes, is also found on chromosome 3. Conversely, chromosome 6 does not contain any gene clusters, underscoring the uneven distribution of these clusters across the genome.

Gene clusters often include members from the same gene subfamily, but some clusters contain genes from different subfamilies. For instance, the cluster containing Capana02g001535, Capana02g001536, and Capana02g001539 consists entirely of genes from the N subfamily. In contrast, the cluster comprising Capana03g004459, Capana03g003360, and Capana03g004466 includes genes belonging to different subfamilies, namely CN, NL, and N, respectively. This diversity within clusters reflects the complexity of the genomic organization and potential functional interactions among these resistance genes. This detailed analysis of NBS-LRR gene distribution, diversity, and clustering provides valuable insights into the genetic architecture and evolutionary dynamics of resistance genes in Capsicum. These findings may facilitate further studies on the functional roles of these genes and their potential applications in breeding programs for disease-resistant crops.

### 2.3. Phylogenetic Analysis of NBS-LRR Resistance Genes in Capsicum: Subfamily Diversity and Evolutionary Insights

**Figure 2:**
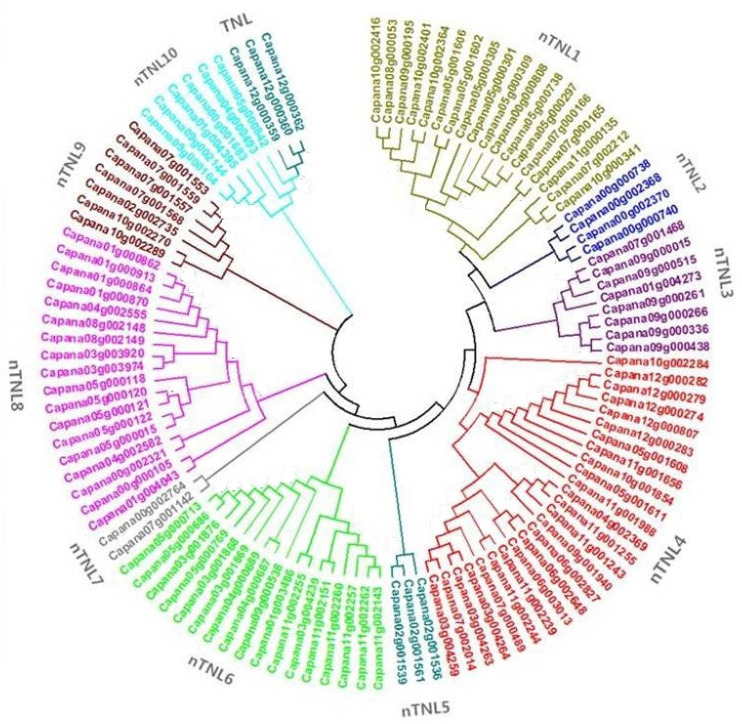
Phylogenetic tree of NBS domains from NBS-encoding genes in pepper (*Capsicum annuum* Zunla-1). The phylogenetic tree was constructed based on 111 aligned amino acid sequences of the conserved NBS domains using the Neighbor-Joining method in MEGA5. The tree is divided into 10 clades of the nTNL subfamily and a single clade of the TNL subfamily. Genes belonging to the same clade are represented in the same color, indicating phylogenetic grouping.

To further explore the phylogenetic relationships of NBS-LRR resistance genes in Capsicum, a phylogenetic tree was constructed using MEGA 5 [24] based on the conserved amino acid sequences of the NBS domains, specifically the region from the P-loop to the GLPL motifs. The phylogenetic tree includes 111 conserved NBS domain sequences, consisting of 108 nTNL genes and 3 TNL genes (Figure 2).

The phylogenetic tree is divided into two distinct clades: one representing members of the nTNL subfamily and the other comprising members of the TNL subfamily. The nTNL subfamily constitutes the majority of the sequences and is further subdivided into 10 smaller clades, designated as nTNL1 through nTNL10. In contrast, the TNL subfamily forms a separate and distinct clade. Each nTNL clade contains a mixture of genes distributed across different chromosomes, indicating a complex genomic distribution.

For instance, the nTNL7 clade contains only two NBS-LRR resistance genes, which are located on different chromosomes: Capana00g002764 is found on chromosome 0, while Capana07g001142 is located on chromosome 7. Similarly, the nTNL9 clade includes seven genes originating from chromosomes 7, 2, and 10, respectively. This mixed chromosomal distribution highlights the genomic diversity within the nTNL subfamily. In contrast, the TNL subfamily clade is relatively simple, containing only three TNL genes, all of which are located on chromosome 12, reflecting a higher level of conservation. This phylogenetic structure not only reveals the distribution characteristics of nTNL and TNL genes in Capsicum but also provides valuable insights into their functional diversity and evolutionary mechanisms.

### 2.4. Structural Diversity of NBS-LRR Resistance Genes in Capsicum: Insights into Domain Arrangements, Subfamily Classification, and Functional Specialization

The identified NBS-LRR resistance genes were categorized into two main groups based on the presence or absence of a TIR domain at the N-terminus: 248 nTNLs (non-TIR NBS-LRR) and 4 TNLs (TIR NBS-LRR). Additionally, the nTNL genes were further classified into 10 subclasses based on the presence or absence of CC and LRR domains: N, NL, NLN, NLL, NN, CN, CNL, CNN, CNLN, and CNLNLN. In contrast, the TNL subfamily was less diverse and contained only one subclass with an unusual domain arrangement, referred to as TN, characterized by the absence of an LRR domain (Table 1). Interestingly, a minority of the identified genes belonged to canonical classes, such as CNLs or TNLs (e.g., Table 1, Class 7). The majority of genes, however, displayed less typical domain arrangements, with most of these genes falling under the nTNL subfamily. This highlights the structural and functional diversity of nTNL genes, which are predominant among the identified NBS-LRR resistance genes.

The nTNL subfamily exhibited significant diversity in domain arrangements. Among its subclasses, the N genes were the most abundant, totaling 172 and accounting for 69.4% of the total identified NBS-LRR resistance genes. Conversely, only 0.8% (2 out of 248) of the nTNL genes were identified as typical CNLs. The remaining subclasses contained comparatively fewer genes, emphasizing the prevalence of atypical domain structures within the nTNL subfamily. The TNL subfamily, in comparison, showed limited diversity, containing only the TN subclass. Notably, an RPW8 NB-ARC (RN) gene was also identified in the pepper genome, located on chromosome 4. The RPW8 gene, known to confer broad-spectrum resistance to powdery mildew in Arabidopsis [15], represents a unique and functionally significant gene within the NBS-LRR family. This classification underscores the structural complexity and diversity of NBS-LRR resistance genes in pepper, with the nTNL subfamily dominating in terms of gene number and domain arrangements. The relatively low number of canonical CNL and TNL genes highlights the evolutionary divergence and potential functional specialization of these genes. These findings provide critical insights into the genomic architecture and evolutionary dynamics of plant immune receptors in Capsicum.

### 2.5. Conserved Motifs in the NBS Domains of Capsicum NBS-LRR Resistance Genes: Structural Insights for Plant Immunity

**Figure 3:**
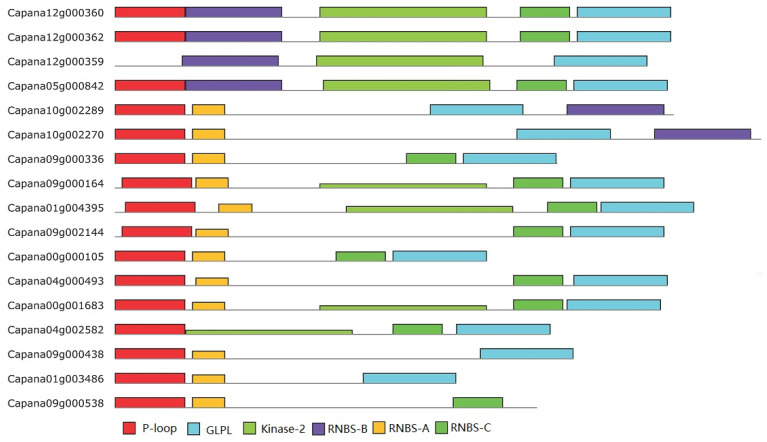
Arrangements of six conserved motifs in NBS-LRR resistance genes of pepper. The figure shows the arrangement of six conserved motifs across different NBS-LRR resistance genes. Each motif is represented by a colored rectangle: red for P-loop, yellow for RNBS-A, light green for kinase-2, purple for RNBS-B, green for RNBS-C, and blue for GLPL. The positions of the motifs correspond to their locations within the amino acid sequences of the respective NBS domains, demonstrating their conserved arrangement across the analyzed genes. These motifs play critical roles in ATP/GTP binding, signal transduction, and structural stabilization in plant immune responses.

Motifs within the NBS domains of the pepper genome were identified using the Multiple Expectation Maximization for Motif Elicitation (MEME) algorithm [25]. Representative genes from each subclass are shown to demonstrate motif arrangements. A total of six conserved motifs were detected, spanning from the N-terminus to the C-terminus of the NBS domains. These motifs include the P-loop, RNBS-A, kinase-2, RNBS-B, RNBS-C, and GLPL motifs (Table 2, Figure 3). These motifs are critical for the function of NBS-LRR resistance genes, as they are associated with ATP binding, nucleotide hydrolysis, and signal transduction during plant immune responses. The P-loop and RNBS-A motifs displayed high similarity across all NBS-LRR resistance gene subclasses, underscoring their evolutionary conservation and essential roles in the functionality of these genes. In contrast, the kinase-2, RNBS-B, RNBS-C, and GLPL motifs exhibited lower levels of conservation (Supplemental Figure S1), indicating potential variations in their functional roles or structural configurations among different gene subclasses.

In the nTNL subfamily, all six motifs were well-conserved, reflecting the functional homogeneity within this group. However, the TNL subfamily displayed greater variability, particularly in the RNBS-A motif. In Class 11 of the TNL subfamily, the RNBS-A motif could not be identified, suggesting that this motif may have undergone significant divergence or loss in certain TNL genes. This observation highlights the distinct evolutionary trajectories and functional diversifications between the nTNL and TNL subfamilies. These findings provide valuable insights into the structural and functional diversity of NBS-LRR resistance genes in pepper. The conserved motifs identified in this study serve as molecular markers for further exploration of gene function and evolutionary relationships. Additionally, the divergence in motif conservation between subfamilies may contribute to the functional specialization of these genes in plant immunity.

## 3. Discussion

### 3.1. Diversity and Evolution of NBS-LRR Resistance Genes in Capsicum and Other Plants

The study of NBS-LRR resistance genes across various plant species has revealed a wide diversity in both the total number of genes and their distribution among subfamilies. *Capsicum* exhibits a total of 252 NBS-LRR resistance genes, including 4 TNLs and 48 CNLs, resulting in a strikingly low TNL-to-CNL ratio of 1:12. This stands in stark contrast to Arabidopsis, where 177 NBS-LRR resistance genes have been identified, with 92 TNLs and 51 CNLs, giving a ratio of nearly 2:1 [26]. Similarly, in *Medicago*, a total of 333 NBS-LRR genes were identified, with a more balanced distribution of 177 CNLs and 156 TNLs [27]. However, in the grape genome, the numbers are significantly smaller, with 60 CNLs and only 19 TNLs identified [28]. These comparisons suggest that the evolutionary trajectories of NBS-LRR resistance genes vary widely across species, shaped by both lineage-specific adaptations and environmental pressures.

One key observation is the notable loss of TNL genes in certain species. In *Capsicum*, the remarkably low number of TNLs compared to CNLs may indicate significant evolutionary losses of the TNL subfamily, potentially as an adaptation to specific environmental conditions. This pattern becomes even more pronounced in monocotyledonous plants, where the TNL subfamily is entirely absent. For example, rice, a representative monocot species, contains 430 regular rice NLR genes with both NBS and LRR domains, 192 belong to the CNL subfamily [29]. Similarly, corn also lacks TNL genes entirely [30]. The complete absence of TNL genes in monocots has been hypothesized to result from gene loss during the divergence of monocots and dicots, allowing monocots to adapt to unique environmental challenges by focusing on the expansion of CNL genes [31]. These findings highlight the contrasting evolutionary patterns between monocots and dicots. In dicots like *Arabidopsis*, *Medicago*, and grape, both TNL and CNL subfamilies coexist, with varying degrees of expansion and contraction, reflecting the lineage-specific adaptations to diverse pathogens. Conversely, monocots exhibit a complete reliance on the CNL subfamily, exhibiting an extreme case of subfamily specialization. This divergence underscores the evolutionary plasticity of NBS-LRR resistance genes and their crucial role in shaping plant immune systems.

### 3.2. Chromosomal Distribution and Evolutionary Expansion of NBS-LRR Resistance Genes in Capsicum

The distribution of NBS-LRR resistance genes across the pepper genome reveals a characteristic uneven pattern, with the number of genes varying among chromosomes. This uneven distribution is not unique to pepper and has also been observed in the genomes of other plants, such as legumes and Brassicaceae species [4,13,18,27,32,33]. Such variability reflects the dynamic genomic rearrangements and evolutionary pressures acting on resistance genes.

A key feature of NBS-LRR resistance genes in pepper is their organization into clusters. Of the 252 identified NBS-LRR resistance genes, 136 are physically clustered, representing 54% of the total, and forming 47 clusters. This clustering pattern is consistent with findings in other plant genomes. For instance, in *Medicago*, 83.6% of NBS-LRR resistance genes are organized into clusters, while in potato, 75% of the NBS-LRR genes are part of 63 clusters, with only 27% classified as singletons [27,34]. Clusters of resistance genes are often associated with adaptive potential, as they can serve as hotspots for generating new mutations and functional diversity [35]. In pepper, Chromosome 12 stands out as having the highest diversity of gene subfamilies within clusters, underscoring its potential role as a hub for resistance gene innovation.

Tandem duplication is a major mechanism driving the expansion and diversification of NBS-LRR resistance genes in pepper. A total of 77 genes represent 30 tandem duplication events, accounting for 31% of the identified genes. Tandem duplications involve two to eight genes located within a genomic range of less than 150 kb, with shorter intergenic distances compared to those in broader gene clusters. Interestingly, all tandem duplications are embedded within larger gene clusters, highlighting their integral role in cluster formation. This mechanism has been widely recognized as a strategy for plants to generate a series of alleles and diversify R-genes under coevolutionary pressures from pathogens [36,37]. The organization and expansion of NBS-LRR resistance genes in pepper provide insights into their evolutionary dynamics and functional adaptability. Clustering and tandem duplication are essential processes that enhance the diversity and versatility of plant immune systems. These findings emphasize the significance of genomic organization in shaping the adaptive potential of resistance genes in *Capsicum* and other plant species.

### 3.3. Phylogenetic Insights into the Evolution of NBS-LRR Resistance Genes in Capsicum

The phylogenetic tree of pepper NBS-LRR resistance genes (Figure 2) is divided into two distinct clades, representing the nTNL and TNL subfamilies. The nTNL clades account for the overwhelming majority of the identified genes, reflecting their dominant role in the evolution of NBS-LRR resistance genes in *Capsicum*. Notably, the five currently known functional NBS-LRR resistance genes in pepper, including *Bs2*, *CaMi*, *Pvr4*, *TSW*, and *L*, all belong to the CNL subfamily. These genes confer resistance to a diverse range of pathogens throughout the pepper life cycle, such as *Xanthomonas campestris* pv. vesicatoria, root-knot nematodes, potyviruses, tomato spotted wilt virus, and *Tobamovirus* spp. [38,39]. This suggests that the evolutionary expansion of CNLs in pepper is likely driven by the need to maintain a broad and diverse spectrum of resistance mechanisms against pathogens.

The nTNL clades exhibit mixed clustering, with genes from different chromosomes grouped together. These mixed clades are indicative of the genomic processes that have shaped the expansion of NBS-LRR resistance genes in pepper, including large-scale genetic recombination and chromosomal rearrangements. Events such as unequal crossing over and gene conversion have played pivotal roles in the loss or gain of resistance genes, driving the generation of pseudogenes as well as novel functional genes [40]. These processes have allowed the pepper genome to continuously adapt to evolutionary pressures, maintaining its ability to respond to diverse biotic stresses.

Genes within the same clade tend to have high sequence similarity, reflecting their recent evolutionary divergence. For instance, the TNL clade contains three tandemly duplicated genes, Capana12g000359, Capana12g000360, and Capana12g000361, located on chromosome 12. These genes exhibit more than 90% sequence similarity, highlighting their shared evolutionary origin and potential functional redundancy or specialization. Tandem duplications such as these are a hallmark of resistance gene evolution, serving as a mechanism for rapid gene family expansion and diversification.

### 3.4. Diversity of the nTNL and TNL Subfamilies

Domain analyses of NBS-LRR resistance genes in the pepper genome reveal that the nTNL subfamily is more diverse than the TNL subfamily. This greater diversity of nTNLs has led to a long-standing hypothesis that the nTNL subfamily is evolutionarily older than the TNL subfamily, as suggested by earlier studies [26,41]. However, recent advances in whole-genome sequencing have provided new perspectives on the origins and evolutionary trajectories of these subfamilies. For instance, Yue et al. conducted a comparative genomic analysis of 38 species, including representatives from eubacteria, archaebacteria, fungi, protists, plants, and animals. Their findings suggest that the TNL subfamily may have an older origin than the nTNL subfamily, challenging previous conclusions [6]. This discrepancy underscores the complexity of the evolutionary history of these gene families, highlighting the need for further analyses and more comprehensive datasets [6].

The numerical dominance of nTNLs over TNLs is a consistent phenomenon across a wide range of angiosperms, indicating a shared evolutionary trend among flowering plants. For example, nTNL genes are the majority in legume species such as soybean (*Glycine max*), common bean (*Phaseolus vulgaris*), and pigeon pea (*Cajanus cajan*) [42]. Similarly, nTNLs predominate in the Cucurbitaceae family, including melon (*Cucumis melo*), watermelon (*Citrullus lanatus*), and cucumber (*Cucumis sativus*) [43]. The same pattern is observed in other plant families, such as Salicaceae (poplar, *Populus trichocarpa*) [44] and Rosaceae (peach, *Prunus persica*, and strawberry, *Fragaria vesca*) [43]. This widespread prevalence of nTNL genes reflects their adaptive significance and potential functional advantages in plant immune systems.

The contrasting evolutionary trajectories of nTNL and TNL genes suggest that their origins and diversification have been shaped by distinct evolutionary pressures. While the numerical dominance of nTNLs points to their role in conferring broad-spectrum resistance and adaptability, the evolutionary persistence of TNLs in some plant lineages indicates their specialized roles in plant defense mechanisms. The divergence in their evolutionary timelines and functional attributes adds to the complexity of understanding the dynamics of plant immune receptor evolution.

### 3.5. Structural Conservation and Divergence in the NBS Domains of Pepper

A total of six conserved motifs were identified within the NBS domains of pepper NBS-LRR resistance genes. These motifs, spanning from the N-terminus to the C-terminus, include the P-loop, RNBS-A, kinase-2, RNBS-B, RNBS-C, and GLPL motifs (Figure 3). Each motif plays a distinct role in the functionality of the NBS domain. For instance, the P-loop and kinase-2 motifs are responsible for binding ATP and GTP, respectively, which are critical for the activation of the NBS domain and subsequent immune signaling [45]. The GLPL motif, also known as the hydrophobic domain, is speculated to function as a transmembrane domain, potentially playing a role in protein localization or interaction [46].

Analyses of motif-specific amino acid sequences reveal key distinctions between the CNL and TNL subfamilies. For example, in the kinase-2 motif, the identity of the terminal amino acid serves as a subfamily marker: tryptophan (W) indicates nTNL genes, while aspartic acid (D) indicates TNL genes. These sequence differences highlight the structural divergence between the two subfamilies, reflecting their specialized roles in plant defense mechanisms.

Compared to other structural domains of NBS-LRR proteins (such as TIR/CC and LRR domains), the NBS domain is the most conserved. This conservation makes it an ideal target for primer design in molecular studies aimed at amplifying resistance genes [13]. Despite its overall conservation, notable differences exist between the NBS sequences of the CNL and TNL subfamilies. In Arabidopsis, for example, the P-loop, kinase-2, RNBS-B, and GLPL motifs exhibit high similarity between the TNL and CNL subfamilies. However, RNBS-C shows moderate similarity, while RNBS-A and RNBS-D exhibit the least similarity [13]. These sequence divergences are beneficial for designing primers to amplify specific resistance genes, enabling researchers to target genes from specific subfamilies or evolutionary lineages. Meantime, genes in clusters (e.g., chromosome 12) or those with atypical domain structures (e.g., NLNLN) are prioritized for knockout/overexpression studies. Additionally, conserved motifs (e.g., kinase-2) serve as targets for mutagenesis to dissect their roles in immune signaling.

In summary, the NBS domain serves as a critical structural and functional element of NBS-LRR resistance genes. While its conserved regions facilitate universal primer design, its subfamily-specific sequence divergences enable precise targeting of CNL and TNL genes. These insights into motif conservation and divergence provide a deeper understanding of the molecular basis of NBS-LRR resistance genes and their evolutionary adaptations.

## 4. Materials and Methods

### 4.1. Identification and Classification of NBS-LRR Genes

The whole genome of pepper (*Capsicum annuum* Zunla-1_v2.0), downloaded from the Plant GARDEN (Genome and Resource Database Entry) (https://plantgarden.jp/en/index, accessed on 1 July 2024), was used to identify the NBS-LRR resistance gene families. The identification process for candidate NBS-encoding genes involved two main steps, as described by [47]. First, BLAST and Hidden Markov Model (HMM) searches were performed using a conserved NBS domain as the query sequence to identify NBS-LRR genes in the pepper genome. For the BLAST search, the expectation value threshold was set to 1.0 [47]. Next, all sequence hits obtained from the initial searches were merged, and redundant sequences were removed using BLAST or HMM searches. The TIR and LRR domains of the identified NBS-encoding genes were analyzed using the InterPro database (https://www.ebi.ac.uk/interpro/, accessed on 15 July 2024). However, the CC domain could not be reliably analyzed using Pfam, as noted in previous studies [48,49]. To address this, CC domains were detected using the COILS program (https://bio.tools/coils, accessed on 18 July 2024) [50] with a threshold of 0.8. All CC domain predictions were subsequently validated through visual inspection.

### 4.2. Chromosomal Mapping of NBS-Encoding Genes and Cluster Assignment

The chromosomal locations of all identified NBS-encoding genes were determined by retrieving annotation data from the Plant GARDEN (Genome And Resource Database Entry). The lengths of each chromosome and the distances between neighboring genes were calculated. Using this information, a genetic linkage map was constructed with MapDraw (Figure 1) [51]. The assignment of gene clusters followed the protocol established for *Medicago truncatula* [27]. If two or more NBS-encoding genes located on the same chromosome were within a 250-kb region, they were considered members of the same gene cluster. Based on this criterion, all identified NBS-encoding genes in the pepper genome were classified as belonging to either cluster loci or singleton loci, and their positions were mapped along the chromosomes.

### 4.3. Phylogenetic Analysis of NBS-Encoding Genes

To investigate the evolutionary relationships of NBS-encoding genes in pepper, a phylogenetic tree was constructed based on the conserved amino acid sequences of the NBS domains, specifically from the P-loop to the GLPL motifs. The conserved NBS domains were identified using BioEdit (version 7.2), and all sequences were aligned with CLUSTALX (version 1.83). Phylogenetic analysis was performed using MEGA5 [24], with trees constructed using the Neighbor-Joining method [52]. The evolutionary history of the taxa analyzed was represented by a bootstrap consensus tree, inferred from 1000 replicates [53]. Evolutionary distances were calculated using the p-distance method [54] and are expressed as the number of amino acid differences per site.

### 4.4. Domains and Motifs Analyses

The TIR and LRR domains of NBS-encoding genes were analyzed using hmmPfam (HMMER) (version 3.1) [55] against the Pfam database [56]. However, the Pfam database could not be used to analyze CC domains, as previously noted [6,48]. Motifs within the NBS domains were identified using the Multiple Expectation Maximization for Motif Elicitation (MEME) algorithm [57].

## 5. Conclusions

This study advances our understanding of the structural and evolutionary dynamics of NBS-LRR resistance genes in pepper. The insights gained here provide a valuable foundation for further research into plant immunity and for developing targeted strategies to enhance disease resistance in crops. Future work should focus on the functional characterization of these genes and their interactions with specific pathogens to unlock their full potential in agricultural applications.

## Figures and Tables

**Table 1:** Consensus sequence of the NBS domains of the CNL and TNL predicted proteins.

Class	Structure	Count	P Loop/kin1a	RNBS-A-non-TIR	RNBS-A-TIR	Kin-2	RNBS-B	RNBS-C	GLPL
CC-NBS-LRR									
1	N	172	GIGKST	VLLEVIGCISNTND	-	KGPRYLVVVDDIWRID	NGSRILLTTRETKVAMYAS	LLNLENGWKLLRDKVF	CQGLPL
2	NL	11	GVGKTT	VVVWVTVPK	-	EKSFLLILDDVWKGIN	SKVIITTRSLEVCRQMR	VTTLNEDESWELFVKNAG	CGGLPLA
3	NLN	7	GAGKTT	IRARCHVSPVYSQRGLLLSLL	-	KRYFILLDDVWDHRAWD	SRILLTTRNNDVAYNV	LRFLTYEESWDLLKLKVFGN	CEGLPL
4	NLL	2	GLGKTT	KIWVCVSDDFDEKR	-	GKRYLLVLDDVWNDD	GASVLATTRLEKVGSIM	SNLSQHDGLLLFMQCAFGQ	CGGVPLA
5	NN	8	GIGKTT		-		SRILLTTSETELAMYA	MNLLNLENCWKLLRDKVFG	CHGLPL
6	CN	37	GLGKTT	KVWVSISQTYD	-	CIIITSRNEDVVKRMG	RLLDDEESWSLFCKVA	LRFLNEEE-	CGGLPLA
								SWDLFCKKLRPE	
7	CNL	2	GVGKTT	LRIWLCASQDFDVTK	-	RGKRFLLIIDDVWSRD	GSKVVVTTRSDYIAAMME	SLKELPHEDCFALF	CGGVPLA
8	CNN	3	GLCKTT		-				CKGLPLA
9	CNLN	5	GVGKTT	DNRELLLEILR	-	GKRFLIVMDDVWDAE	GSRILLTTRSDDVAQY	LRPLGEEDSWILLEKKIFQQ	KTKGLPF
10	CNLNLN	1	GLGKTT	RAWCYVSEVYKNKELLLEIL	-	KRKRYLIVLDDLWDIK	GSRILVTSRLQHLASQV	LRFFTVEESWELLQKKVF	CHGLPL
TIR-NBS-LRR									
11	TN	4	GIGKTE	-		RWKKVLFILDDVNHRE	GSRIILTARDRHL	VQLLSEDEALELSSRHAF	AGGLPL

**Table 2:** Six conserved motifs in the NBS domains of pepper (*Capsicum annuum*).

Name	Domains	Motifs	Width	Motif Similarity Matrix					
				P-loop	GLPL	Kinase-2	RNBS-B	RBAS-A	RNAS-C
MEME-1	P-loop	GJGKTTLARKVYNDP	15	-	0.19	0.23	0.21	0.21	0.26
MEME-2	GLPL	CPPELEEIGKZIAKKCGGLPL	21	0.19	-	0.13	0.22	0.16	0.2
MEME-3	Kinase-2	RYLIVLDDVWSTDAW	15	0.23	0.13	-	0.2	0.3	0.22
MEME-4	RNBS-B	NGSRIJLTTRNEEVA	15	0.21	0.22	0.2	-	0.24	0.16
MEME-5	RBAS-A	SHFDIRAWVTVSQEYNRRELL	21	0.21	0.16	0.3	0.24	-	0.18
MEME-6	RNAS-C	LSEEESWKLLRKKVF	15	0.26	0.2	0.22	0.16	0.18	-

## Data Availability

The original contributions presented in this study are included in the article/Supplementary Material. Further inquiries can be directed to the corresponding author.

## Supplementary Materials

The supporting information can be downloaded at: https://www.mdpi.com/article/10.3390/ijms26051828/s1.
