# Supplementary material for: Phylogenetic, Structural, and Evolutionary Insights into Pepper NBS-LRR Resistance Genes
Source: Int J Mol Sci. 2025 Feb 20;26(5):1828. doi: 10.3390/ijms26051828 (PMC11899730; doi:10.3390/ijms26051828)
Supplement: Supplementary file 1 [file ijms-26-01828-s001.zip › ijms-3470211-supplementary.pdf]

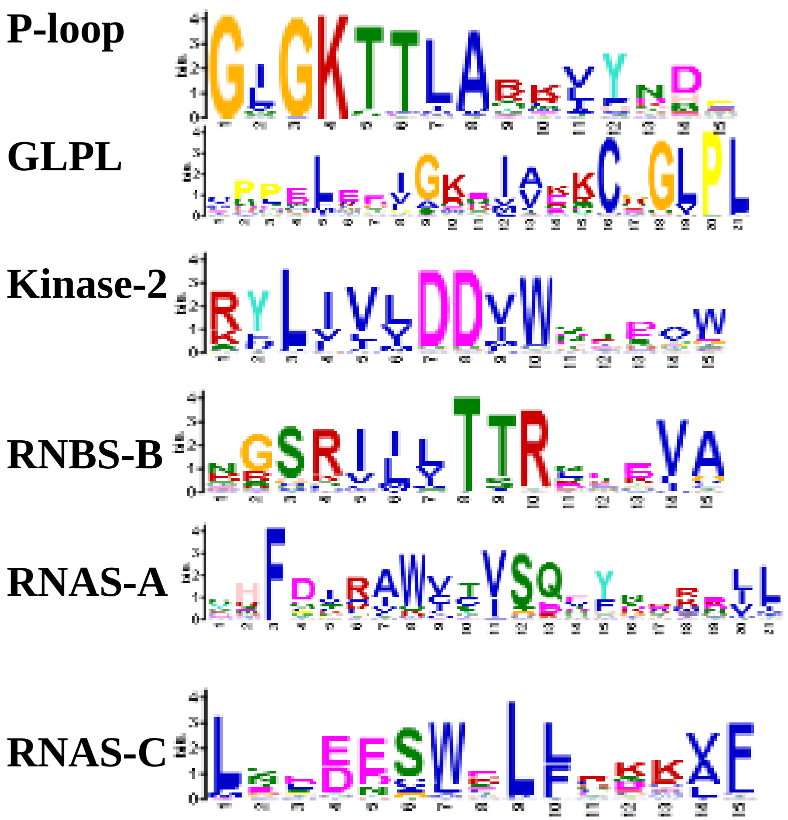

Supplemental Figure S1 Conserved motifs in the NBS domains of pepper (*Capsicum annuum*) were identified using the MEME tool. The identified motifs, listed from the N-terminus to the C-terminus, include P-loop, GLPL, kinase-2, RNBS-B, RNBS-A, and RNBS-C. Name of each motif is shown on the left side of its corresponding sequence logo.
